# Morphological differentiation of *Brachionus calyciflorus* caused by predation and coal ash pollution

**DOI:** 10.1038/s41598-017-16192-w

**Published:** 2017-11-17

**Authors:** Ying-Hao Xue, Xiao-Xue Yang, Gen Zhang, Yi-Long Xi

**Affiliations:** 1 Provincial Key Laboratory for Conservation and Utilization of Important Biological Resource in Anhui, College of Life Sciences, Anhui Normal University, Wuhu, Anhui 241000 China; 2 Rural Energy & Environment Agency, Ministry of Agriculture, Beijing, 100125 China; 3 Shenzhen GenProMetab Biotechnology Co., Ltd, Shenzhen, Guangdong 518101 China; 4 Collaborative Innovation Center of Recovery and Reconstruction of Degraded Ecosystem in Wanjiang City Belt, Anhui Province, Anhui Normal University, Wuhu, Anhui 241000 China

## Abstract

Different rotifer stains exhibited remarkably morphological differences which could not be eliminated under laboratory conditions. In the present study, we hypothesized that predation pressure and pollution might be two forces driving morphological differentiation of rotifer. To test this hypothesis, rotifers (*Brachionus calyciflorus*) belonging to two sibling species were collected from three special lakes (with coal ash pollution, high predation pressure or neither) and cultured for more than three months to investigate their potential differentiation in morphology. Twelve morphological parameters were measured and compared among three lakes at four food density (*Scenedesmus obliquus*). The results showed that most of the tested morphological parameters changed in response to food level and differed among three habitats. Rotifers from the habitat with high predation pressure evolved stable long posterior lateral spine and relatively small body size. Rotifers collected from the polluted habitat was of smaller body size, compared with those from ordinary habitat. Bigger eggs were laid by rotifers from polluted area or lake with high predation pressure, enabling newborns more resistant to pollution or predation, and thus ensuring the survival rate of newborns. Finally, we concluded that both predation and pollution could affect the morphological differentiation and evolution of rotifers.

## Introduction

Phenotypic plasticity is an important factor determining the spatial and temporal distribution of zooplankton species^1^. Rotifers are an important link between phytoplankton and carnivorous zooplanktons and fishes in aquatic food web^2^. They are of great commercial and scientific interests^3^ and are model organisms to study environmentally induced morphological variations^4^.

Laboratory assays revealed that various environmental factors affected the morphological characters of rotifers, such as food type^5^, food quality^6^ and food density^7^, temperature^8^, salinity^4^, desiccation^9,10^ and predation^11,12^. Generally, the morphological variations are adaptive responses to environmental changes. There is no doubt that rotifers living in different environments differ in morphology. However, when these rotifers were transferred to the same cultural conditions in laboratory, the morphological differences still exist. For example, Snell and Carrillo^13^ studied the effects of temperature and salinity on the morphological characters of 13 geographic *Brachionus plicatilis* strains and the results showed that the strain was the most important fact determining rotifer morphology. These results suggested that after long term adaptation to local environments, rotifer might evolve stable morphological characters.

Up to date, a few studies have investigated the ecological mechanisms underlying the morphological differentiation from various environments. After cultured under laboratory conditions for several months, *Brachionus calyciflorus* showed markedly larger body size and egg size in Guangzhou (China) strain than those in Wuhu (China) strain^5^, suggesting that temperature might be a natural factor to select the morphology of rotifers. If this opinion was correct, whether other environmental factors could also potentially drive the morphological differentiation remains unknown. To resolve this question, more investigations should be conducted on rotifers from various environments.

Certain zooplankton predators, such as predatory copepods and the rotifera *Asplanchna*, could induce phenotypic plasticity of prey rotifers, thereby altering their morphology, physiology, life history or behavior^14,15^. Both laboratory and field studies demonstrated that loricate rotifera exhibit strong morphological defenses in response to predation pressure^16–20^. Thus, predation pressure might act as natural selection on the morphology of rotifer. Moreover, Hanazato and colleague^21–23^ reported that the morphology of *Daphnia* changed in response to pollution, suggesting that environmental pollution was probably another driving force during the morphological differentiation.

Food is one of the most important factor affecting the morphology of zooplanktons^24^. Fed on *Chlorella pyrenoidosa*, *Brachionus angularis* showed enlarged body size with increasing food level with the egg volume larger at intermediate food level^25^; the body size and egg size of *B. calyciflorus* increased linearly with increasing food level^26^. In natural waterbodies, the density of algae varies among lakes and seasons. While investigating the effects of other environmental factors on the morphology of zooplankton, the level of algal food should be considered.

During 2009, our group collected and clonally cultured the rotifer *B. calyciflorus* from three lakes located in Wuhu City, China (Lake Hui, Lake Fengming and Lake Tingtang; Supplementary Figure 1) to compare their genetic structure and life history parameters^27–29^. These three lakes were all supplemented from the Yangtze river, but their environmental factors were quite different. Lake Hui was polluted by coal ash, exhibiting a low level of alga and a high level of heavy metal contaminations. Lake Fengming had a high level of predation on *B. calyciflorus*. Lake Tingtang is a public park and no obvious pollution or high level of predation on *B. calyciflorus* was observed. The clonally cultured rotifers from these three lakes provided an opportunity to investigate the selective effects of predation and pollution on the morphological characteristics of rotifers.

In the present study, to test the potential selection of pollution and predation on the morphological characteristics of the rotifer *B. calyciflorus*, rotifers for Lake Tingtang, Lake Fengming and Lake Hui were cultured at different food concentrations. Their morphological characteristics were determined and compared. The results would partially explain the morphological differentiation of rotifers in fields and contribute basic data to the mechanisms underlying morphological evolution.

## Materials and Methods

### Ethics Statement

No specific permit is needed for rotifer studies in P. R. China. The location at which the rotifers were collected does not belong to any national parks, protected areas or private lands. There were no protected species in the sampling areas, and no local laws or regulations were overlooked.

### Study area

Lake Hui (31°16′12′′N, 118°12′36′′E), Lake Fengming (31°24′55′′N, 118°23′27′′E) and Lake Tingtang (31°21′49″N, 118°22′50″E) are all located in Wuhu City, China, south to the Yangtze River (Supplementary Figure 1). The average depth of these three lakes was 6 m, 1.2 m and 1.5 m, respectively, with the water area of 21.12 ha, 40.27 ha and 13.47 ha, respectively. Lake Hui is a water pond to store coal fly ash discharged by the Wuhu thermal power station. It was polluted by As, B, Mo, Se and V, and showed relatively higher level of pH, Al, Ca, Cr, K, Li and Si, with the lower level of Chl-a and total dissolved nitrogen (TDN), compared with other two lakes. Lake Fengming is used to culture fishes and high density of copepods and Asplanchna was observed at the time for sampling. The background chemical conditions of these three lakes were listed in Table S1.

### Rotifer collection and identification of sibling species

Rotifers collected from these three lakes were clonally cultured at 22 °C at a natural light for more than 3 months. They were cultured in 10-ml glass test tubes and fed on *Scenedesmus obliquus* (2.0 × 10^6^ cells/ml) every day, and the rotifer medium^30^ was renewed twice a week. The alga was growing in semi-continuous culture using HB-4 medium^31^. Alga cells were concentrated by centrifugation, resuspended in the rotifer culture medium and counted using a hemocytometer before feeding.

Based on the analyses of rDNA 18S–28S ITS and mtDNA COI fragments, rotifers were classified into two sibling species^27,28^. The sibling species I was observed in all the three lakes, but sibling species II was only found in Lake Tingtang. Thus, only sibling specie I was used in the present study. A total of 28 *B. calyciflorus* clones belonging to sibling species I were randomly selected for the present study, including 10, 9 and 9 clones from Lake Hui, Lake Fengming and Lake Tingtang, respectively. Each clone was cultured and determined separately.

### Rotifer culture at different food concentrations

For each clone, four food concentrations were prepared, including 0.75 × 10^6^, 1.5 × 10^6^, 3.0 × 10^6^ and 6.0 × 10^6^ cells/ml of *S. obliquus*. Rotifers were cultured for 10 days. During this period, the culture medium was changed at 50% and then fed with fresh alga daily. Next, rotifers were observed under a dissecting microscope and neonates could be recognized based on the size. More than 100 neonates were randomly transferred from each clone to a new tank using a glass pipette and further cultured at the same conditions. The neonates were checked every 2 h. Once the first amictic egg was laid, the neonate was fixed in 4% formaldehyde solution.

### Morphometry

**Figure 1:**
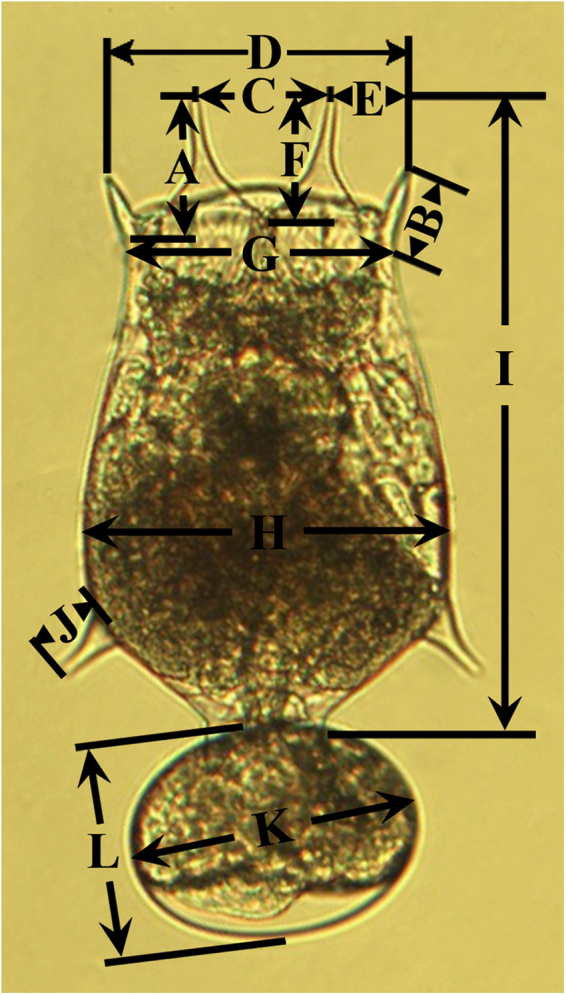
*Brachionus calyciflorus* showing the measured morphological characters. (**A**) Anterior medial spine length; (**B**) anterior lateral spine length; (**C**) distance between anterior medial spines; (**D**) distance between anterior lateral spines; (**E**) distance between anterior lateral and medial spines; (**F**) dorsal sinus depth; (**G**) head aperture; (**H**) lorica width; (**I**) lorica length; (**J**) posterior lateral spine length; (**K**) egg major diameter; (**L**) egg short diameter.

Based on Fu *et al*.^32^ and Ciros-Pérez *et al*.^33^, 12 morphological indices were measured, including anterior medial spine length, anterior lateral spine length, distance between anterior medial spines, distance between anterior lateral spines, distance between anterior lateral and medial spines, dorsal sinus depth, head aperture, lorica width, lorica length, posterior lateral spine length, egg major diameter and egg short diameter (Fig. 1). For each treatment, 30 fixed amictic egg-bearing females were randomly selected and measured. Symmetrical structures, containing anterior medial spine, anterior lateral spine and posterior lateral spine (only for the morphotype with two posterior lateral spines), were both measured and the average values were used for statistical analyses.

Body size (V_body_) was calculated by V_body_ = 0.2a^2^b, where a and b is the length and width of lorica, respectively. Egg size (V_egg_) was calculated using the formula: V_egg_ = (4/3)π(a^2^b + ab^2^)/16, where a and b are the two diameters of ellipsoid egg^7^.

### Statistical analyses

All data were tested for normality using the one-sample Kolmogrov–Smirnov procedure. Homogeneity of variances was checked using the Levene’s test. Two-way analyses of variance were conducted to identify significant effects of food concentration, sampling lake and their interaction on each morphological parameter. One-way analyses of variance was performed to determine the effects of food level on morphology of rotifers. Considering all the 12 tested morphometric characters, Wilk’ Lambda step-wise discriminant analysis was conducted among three lakes. Data analysis was performed using the SPSS software (release 11.5).

## Results

### Correlation of biometric parameters with food density

The results of biometric parameters in each population at four food concentrations are listed in Tables 1 and 2. All the 12 morphological characters were significantly decreased at a high food density (6.00 × 10^6^ cells/mL), compared with those at moderate or low algal densities, except posterior lateral spine length from Lake Tingtang (SNK tests).

**Table 1:** Effects of food density on the morphological indices of *Brachionus calyciflorus* spines collected from three lakes.

Parameters	Food density (cells/mL)	Lake Fengming	Lake Hui	Lake Tingtang
Anterior medial spine length (μm)	0.75 × 10^6^	41.6 ± 0.7^a^	42.4 ± 0.7^c*^	46.9 ± 0.5^a^
	1.50 × 10^6^	40.4 ± 0.4^a^	40.3 ± 0.6^a*^	45.3 ± 0.5^c^
	3.00 × 10^6^	40.7 ± 0.4^a^	43.4 ± 0.5^c*^	45.1 ± 0.5^c^
	6.00 × 10^6^	36.8 ± 0.8^b^	38.3 ± 0.4^b*^	43.4 ± 0.4^b^
Anterior lateral spine length (μm)	0.75 × 10^6^	34.4 ± 0.5^a^	34.4 ± 0.5^c*^	37.9 ± 0.4^a^
	1.50 × 10^6^	35.4 ± 0.5^a^	33.0 ± 0.5^a*^	36.0 ± 0.5^b^
	3.00 × 10^6^	34.0 ± 0.5^a^	35.4 ± 0.4^c*^	36.4 ± 0.4^b^
	6.00 × 10^6^	30.1 ± 0.6^b^	31.1 ± 0.4^b*^	34.5 ± 0.4^c^
Distance between anterior medial spines (μm)	0.75 × 10^6^	42.9 ± 0.8^a^	42.1 ± 0.6^a^	43.6 ± 0.6^a^
	1.50 × 10^6^	43.4 ± 0.6^a^	41.0 ± 0.6^a^	42.4 ± 0.7^a^
	3.00 × 10^6^	42.1 ± 0.7^*^	41.7 ± 0.6^a^	40.2 ± 0.6^b^
	6.00 × 10^6^	40.3 ± 0.7^b*^	39.2 ± 0.4^b*^	43.1 ± 0.6^a^
Distance between anterior lateral spines (μm)	0.75 × 10^6^	119.9 ± 1.1^c*^	119.9 ± 1.1^a*^	123.7 ± 0.8^a^
	1.50 × 10^6^	119.4 ± 0.7^c^	115.6 ± 1.0^b*^	120.0 ± 0.8^b^
	3.00 × 10^6^	114.9 ± 0.7^a^	119.4 ± 0.8^a*^	116.8 ± 0.9^c^
	6.00 × 10^6^	110.6 ± 1.7^b^	110.5 ± 0.6^c^	113.5 ± 0.8^d^
Distance between anterior lateral and medial spines (μm)	0.75 × 10^6^	39.4 ± 0.4^a*^	39.8 ± 0.4^a*^	40.9 ± 0.3^a^
	1.50 × 10^6^	38.9 ± 0.3^a^	38.4 ± 0.4^b*^	39.8 ± 0.4^b^
	3.00 × 10^6^	37.3 ± 0.4^b*^	39.7 ± 0.4^a*^	39.2 ± 0.4^b^
	6.00 × 10^6^	35.9 ± 0.6c	36.4 ± 0.3^c^	36.0 ± 0.3^c^
Dorsal sinus depth (μm)	0.75 × 10^6^	46.9 ± 0.7^a*^	49.8 ± 0.7^bc*^	52.1 ± 0.5^a^
	1.50 × 10^6^	46.3 ± 0.5^a*^	48.2 ± 0.7^b*^	51.1 ± 0.6^a^
	3.00 × 10^6^	46.5 ± 0.5^a*^	51.1 ± 0.6^c^	51.2 ± 0.6^a^
	6.00 × 10^6^	43.4 ± 0.9^b*^	46.0 ± 0.4^a*^	49.3 ± 0.5^b^
Head aperture (μm)	0.75 × 10^6^	115.4 ± 0.9^a*^	114.3 ± 1.0^bc*^	117.4 ± 0.6^a^
	1.50 × 10^6^	113.3 ± 0.5^a^	112.3 ± 0.8^b^	116.6 ± 0.7^a^
	3.00 × 10^6^	113.1 ± 0.5^a*^	115.7 ± 0.7^c^	115.7 ± 0.8^a^
	6.00 × 10^6^	107.5 ± 1.6^b*^	108.2 ± 0.5^a*^	112.0 ± 0.5^b^
Posterior lateral spine length (μm)	0.75 × 10^6^	41.8 ± 1.3^bc*^	35.9 ± 0.9^a^	37.5 ± 1.0^a^
	1.50 × 10^6^	44.3 ± 1.3^b*^	33.0 ± 1.2^bc^	34.5 ± 1.0^b^
	3.00 × 10^6^	39.8 ± 1.3^c*^	33.8 ± 0.8	34.5 ± 1.1^b^
	6.00 × 10^6^	34.7 ± 0.8^a^	31.6 ± 0.7^c*^	36.2 ± 0.9

Letters indicate sample means that are similar (same letter) or different (different letter) for each variable among different food levels. * represents significantly different from sibling species I from Lake Tingtang.

**Table 2:** Effects of food density on the morphological indices of *Brachionus calyciflorus* lorica and egg collected from three lakes.

Parameters	Food density (cells/mL)	Lake Fengming	Lake Hui	Lake Tingtang
Lorica width (μm)	0.75 × 10^6^	156.7 ± 0.9	151.5 ± 1.1^a*^	159.2 ± 0.7
	1.50 × 10^6^	158.5 ± 0.7	153.2 ± 0.9^a*^	160.6 ± 0.8^a^
	3.00 × 10^6^	159.3 ± 0.7^a^	159.3 ± 0.8^b^	158.2 ± 0.9^b^
	6.00 × 10^6^	155.1 ± 2.2^b^	155.8 ± 0.7^c^	158.0 ± 0.7^b^
Lorica length (μm)	0.75 × 10^6^	245.5 ± 1.8^a*^	241.6 ± 2.0^b*^	255.8 ± 1.2^a^
	1.5 × 10^6^	249.6 ± 1.2^a*^	239.8 ± 1.6^bc*^	255.1 ± 1.4^a^
	3.00 × 10^6^	245.7 ± 1.1^a^	247.9 ± 1.4^a^	247.9 ± 1.4^b^
	6.00 × 10^6^	235.1 ± 3.5^b*^	236.7 ± 1.0^c*^	246.0 ± 1.2^b^
Egg major diameter (μm)	0.75 × 10^6^	120.6 ± 0.8^a*^	120.9 ± 0.9^a*^	117.2 ± 0.6^a^
	1.50 × 10^6^	122.6 ± 0.7^a*^	116.7 ± 0.9^c^	116.3 ± 0.8^a^
	3.00 × 10^6^	116.5 ± 0.7^b*^	117.6 ± 0.7^c*^	113.7 ± 0.7^b^
	6.00 × 10^6^	112.8 ± 1.6^c^	111.7 ± 0.6^b^	111.4 ± 0.6^c^
Egg short diameter (μm)	0.75 × 10^6^	87.1 ± 0.5^a*^	86.6 ± 0.7^a*^	83.0 ± 0.3^a^
	1.50 × 10^6^	87.1 ± 0.3^a*^	84.8 ± 0.5^b*^	82.6 ± 0.5^a^
	3.00 × 10^6^	83.8 ± 0.4^b*^	82.7 ± 0.5^c*^	81.2 ± 0.5^b^
	6.00 × 10^6^	80.6 ± 1.2^c^	78.2 ± 0.3^d^	78.9 ± 0.4^c^

Letters indicate sample means that are similar (same letter) or different (different letter) for each variable among different food levels. * represents significantly different from sibling species I from Lake Tingtang.

**Figure 2:**
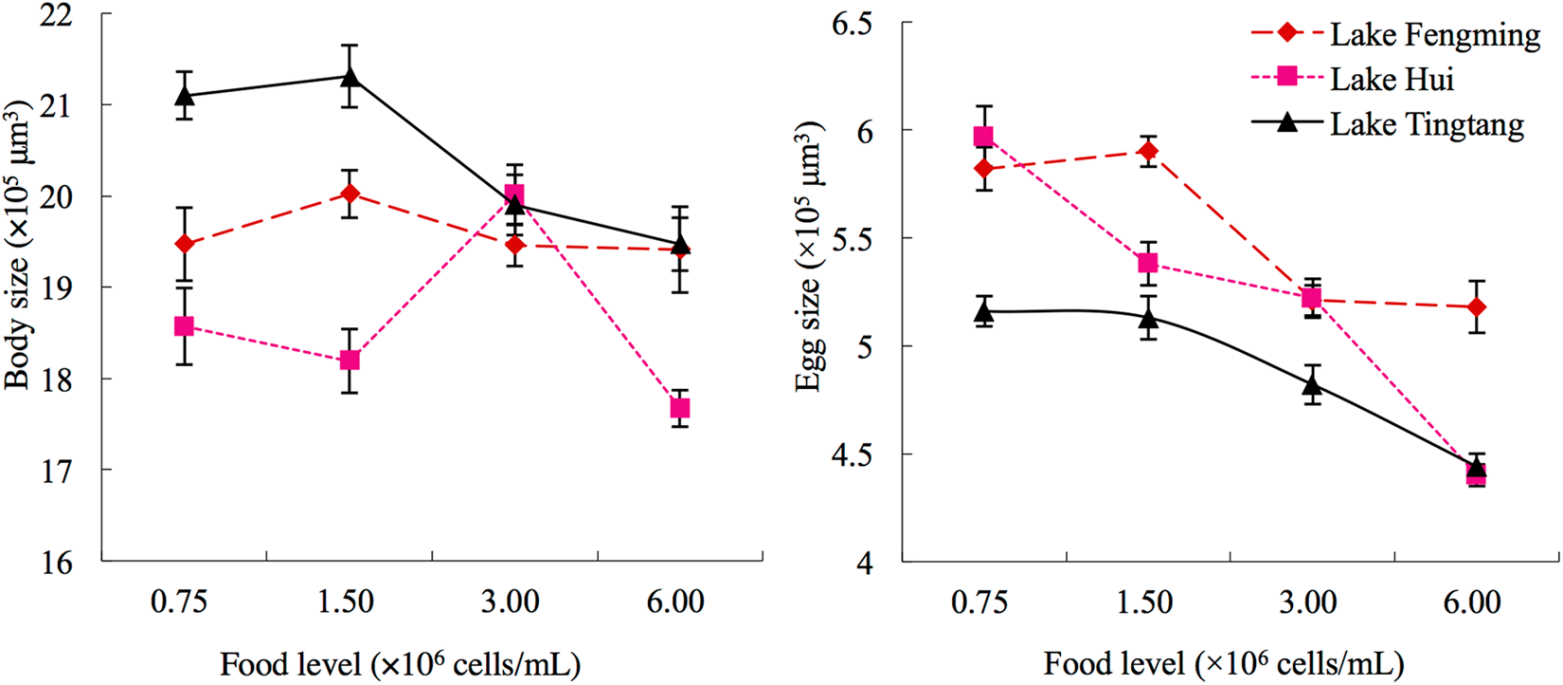
Body size and egg size (×10^5^ μm^3^) of *Brachionus calyciflorus* sibling species I from three lakes at four algal densities (×10^6^ cells/mL).

Among treatments with different food concentrations, the largest body size of rotifers from Lake Hui and Lake Tingtang were observed at 3.00 × 10^6^ cells/mL and low food concentrations (0.75 × 10^6^ and 1.50 × 10^6^ cells/mL), respectively. The body size of rotifers from Lake Fengming did not significantly change in response to food concentration (Fig. 2). Along with descending algal density, the egg size of all the three groups reduced significantly (Fig. 2).

### Effects of coal ash pollution on the morphological characters of rotifers

The values of tested 10 morphological characters of rotifers from Lake Hui, including anterior medial spine length, anterior lateral spine length, distance between anterior medial spines, distance between anterior lateral spines, distance between anterior lateral and medial spines, dorsal sinus depth, head aperture, lorica width, lorica length and posterior lateral spine length, were all shorter or similar, compared with those from Lake Tingtang (Tables 1 and 2). In comparison, the egg major and short diameters of rotifers from Lake Hui were higher than or similar to those from Lake Tingtang (Table 2).

The body size of rotifers from Lake Tingtang was bigger than that from Lake Hui at 1.50 × 10^6^ and 6.00 × 10^6^ cells/mL. At other food levels, the body size was similar between these two populations (Fig. 2).

Rotifers from Lake Hui showed bigger egg size than Lake Tingtang at both 0.75 × 10^6^ cells/mL and 3.00 × 10^6^ cells/mL, but similar to that from Lake Tingtang at 1.50 × 10^6^ cells/mL and 6.00 × 10^6^ cells/mL (Fig. 2).

### Effects of predation on the morphological characteristics of rotifers

The morphological parameters of rotifers were compared between Lake Fengming and Lake Tingtang. No significant differences were observed in anterior medial spine length, anterior lateral spine length and lorica length. The distance between anterior medial length was higher in Lake Fengming than in Lake Tingtang at 3.00 × 10^6^ cells/mL, but lower at 6.00 × 10^6^ cells/mL. Distance between anterior lateral spines, distance between anterior lateral and medial spines, dorsal sinus depth, head aperture, lorica length were shorter in Lake Fengming in comparison to Lake Tingtang at some food levels. Notably, the posterior lateral length, egg major and short diameters were all higher in Lake Fengming compared with Lake Tingtang at 0.75 × 10^6^ cells/mL, 1.50 × 10^6^ cells/mL and 3.00 × 10^6^ cells/mL, but similar at 6.0 × 10^6^ cells/mL (Tables 1 and 2).

Rotifers from Lake Tingtang showed larger body size than those from Lake Fengming at 0.75 × 10^6^ cells/mL, but they produced smaller eggs at all tested food concentrations.

Two-way ANOVA among three lakes were conducted at four food densities. The results stated that body size, egg size and 12 morphometric indexes were all significantly affected by food density, lake and their interaction except head aperture and dorsal sinus depth which were not influenced by lake × food density interaction (Table 3).

**Table 3:** Effects of food density, lake and their interaction on the morphometric indices of *Brachionus calyciflorus* (*F* value).

Indexes	Lake	Food density	Lake × Food density
Degree of freedom	2	3	6
Posterior lateral spine length	90.28**	16.54**	4.29**
Anterior medial spine length	96.06**	32.52**	2.65*
Anterior lateral spine length	45.77**	39.71**	5.39**
Distance between anterior medial spines	5.68**	6.16**	4.29**
Distance between anterior lateral spines	7.20**	54.83**	4.44**
Distance between anterior lateral and medial spines	8.40**	57.98**	3.72**
Dorsal sinus depth	72.62**	21.40**	1.69
Head aperture	17.14**	36.93**	1.97
Lorica width	17.48**	5.80**	5.50**
Lorica length	35.69**	18.60**	5.67**
Egg major diameter	17.13**	48.89**	4.31**
Egg short diameter	34.06**	82.45**	3.33**
Egg size	66.07**	46.67**	6.97**
Body size	5.98**	31.11**	5.64**

*P < 0.05; **P < 0.01.

To recognize rotifers from different lakes, step-wise discriminant analysis was conducted using biometric values at each food level. Unfortunately, none discriminant function was obtained with misclassification error below 40%.

## Discussion

### Morphological changes in response to food level

In the present study, the threshold concentration was 3.0 × 10^6^ cells/mL for rotifers from Lake Hui, and it ranged from 0.75 × 10^6^ cells/mL to 1.5 × 10^6^ cells/mL for rotifers from Lake Tingtang. The largest average value of body size emerged at 1.5 × 10^6^ cells/mL for rotifers from Lake Fengming, although no statistical differences were observed. This discrepancy might be attributed to the different trophic levels and food concentrations in their original habitats. The concentrations of TDN and Chl-a were lower in Lake Hui, and the levels of heavy metal pollutants were higher, compared with Lake Fengming and Lake Tingtang. Rotifers from Lake Hui might invest more energy to increase body size for resistance of starvation and pollution even at 3.0 × 10^6^ cells/mL food level. These results suggested that the special environments with limited food but high pollution might select rotifers with relatively large body size.

In the present study, relatively bigger egg size was observed at low food levels (0.75 × 10^6^ and 1.5 × 10^6^ cells/mL) for rotifers from all the three lakes. Along with increasing food level, the frequency of egg laying becomes higher. Deposition of more eggs results in offspring of smaller size^34^.

### Effects of predation on the morphology of rotifers

In brachionid rotifers, polymorphism consists of variations in spine length, generally associated with changes in body size^35,36^. In several species of this family, the development of spines is induced by chemical factors released from predators or competitors^37^. *B. havanaensis* responded to the presence of *Asplanchna* by increasing its body length and posterior spine length^38^. Similar results were observed in *Keratella americana*, *K. cochlearis* and *K. tropica* ^18^. Long spines could be strong deterrents against predators^37^. In the present study, Lake Fengming exhibited high predation pressure on *B. calyciflorus*. Offsprings produced by rotifers collected from Lake Fengming developed longer posterior lateral spines, in comparison to those from Lake Tingtang, although they had been cultured for more than three months in the absence of predators. These results indicated that high predation pressure might effectively select rotifers with long posterior lateral spines.

To explain the changes of prey body size, Brooks & Dodson^39^ raised the size-efficiency hypothesis. Generally, planktonic herbivores compete for fine particulate matters. Larger zooplankters do so more efficiently and can take larger particles. When predation is of low intensity, small planktonic herbivores will be competitively eliminated by large forms. In comparison, when predation is intense, size-dependent predation will eliminate the large forms, allowing the small zooplankters that escape predation to become the dominants^39^. Sarma & Nandini^40^ found that small preys (*Anuraeopsis fissa*) could coexist with large predators (*Asplanchna brightwellii* and *A. sieboldi*) since smaller size resulted in lower encounter and consumption rates. In the present study, the body size of sibling species I from Lake Fengming was significantly smaller than those from Lake Tingtang, which could be explained by the size-efficiency hypothesis. Large size of *B. calyciflorus* in Lake Fengming might be predated and small forms survived finally. In addition, rotifers from Lake Fengming possessed long posterior lateral spines which required more energy consumption, and might consequently decrease their body size.

Due to low efficiency of food-collection, small rotifers would increase the metabolic demands per unit mass and permit less assimilation to support egg production^39^. In the present study, rotifers from Lake Fengming should have relatively lower efficiency of food filtering due to their small body size. Thus, the energy investment to reproduction should decrease. The development of long posterior lateral spines further reduced the energy input to reproduction. Finally, predation pressure changed the trade-off of energy between reproduction and growth of preys, number of eggs and egg size. The number of offspring reduced, and thus the egg was enlarged for rotifers from Lake Fengming.

### Effects of coal ash pollution on the morphology of rotifers

Small species are more resistant to anthropogenic stresses^41,42^. Thus, chronically stressed environments select smaller organisms^43^. Cattaneo *et al*.^43^ found that the size distribution of diatoms, camoebians and cladocerans shifted to smaller individuals in a lake polluted by heavy metals and acid. Pesticides also induced the dominance by small species^44^. Persson^45^ investigated long-term liming pollution and the results showed that the average size of the whole community was significantly smaller in the polluted lakes, compared with clean areas.

In the present study, Lake Hui was historically polluted by coal ash, which showed a high pH value, typical metal pollutants (Al, As, B, Cr, Mo, Sb, Se and V) and low algal density. Rotifers have high species diversity, containing both tolerant and sensitive taxa and individuals^23^. Large species are more sensitive to chemicals than small ones^21,44,46^, as organisms need energy to resist environmental stresses and small individuals require less energy to survive^47^. Moreover, rotifers with larger size show a lower growth rate and a longer reproductive period, lifespan and mean generation time in comparison to smaller-sized rotifers^34^. Under extreme conditions, fast reproduction help rotifers survive^48^ and large-sized rotifers with lower growth rate will be eliminated during intraspecific competitions. In the present study, rotifers from Lake Hui were smaller than sibling species I from Lake Tingtang at a low food level, suggesting that coal ash pollution might select small-sized *B. calyciflorus*. Similar body sizes were observed between rotifers from Lake Tingtang and Lake Hui at 3.0 × 10^6^ cells/mL, probably due to their different responses to the changes of food level.

Egg size is a crucial element of life history. Larger eggs often have higher fitness than smaller ones^49^, as they can produce higher quality neonates, which showed a longer starvation time^50,51^ and higher tolerance to chemicals^44^. In the present study, different from body size, the eggs of rotifers from Lake Hui were significantly bigger than those from Lake Tingtang at both 0.75 × 10^6^ and 3.0 × 10^6^ cells/mL. The aggrandized eggs in Lake Hui might result from the reduced investment to reproduction and big eggs in Lake Hui might be morphological adaptation to low food level and heavy metal contaminations.

In the present study, we only investigated three lakes and there were no data to support whether high heavy metal concentrations or low food level was the dominant factor affecting the morphology of rotifers in Lake Hui. Further investigations are required to explore the underlying mechanism in future.

## Conclusions

In the present study, rotifers were originally collected from habitats polluted by coal ash, with high predation pressure or neither, and their morphological responses to food density were compared. As the results indicated, the body size and egg size of rotifers were a unimodal response to food level. Under high predation pressure, rotifers with small body size and long posterior lateral spines would be retained after natural selection. Large-sized rotifers could not compete with small-sized individuals in coal-ash polluted environments and the body size of survived rotifers became smaller. The egg size of rotifers from habitats polluted by coal ash or with high predation pressure was significant larger than those from normal habitat, probably due to the reduced investment to reproduction and subsequently decreased clutch size. Taken together, the present results suggested that high predation pressure or coal ash pollution would drive the natural selection on the morphology of rotifers.

## Electronic supplementary material

Supplementary information
