# Supplementary material for: Morphological differentiation of Brachionus calyciflorus caused by predation and coal ash pollution
Source: Sci Rep. 2017 Nov 17;7:15779. doi: 10.1038/s41598-017-16192-w (PMC5693999; doi:10.1038/s41598-017-16192-w)
Supplement: Supplementary file 1 — Supplementary information [file 41598_2017_16192_MOESM1_ESM.pdf]

Morphological differentiation of *Brachionus calyciflorus* caused by predation and coal ash pollution

Ying-Hao Xue[1,2#], Xiao-Xue Yang[3#], Gen Zhang[3], Yi-Long Xi[1,4*]

1. Provincial Key Laboratory for Conservation and Utilization of Important Biological Resource in Anhui, College of Life Sciences, Anhui Normal University, Wuhu, Anhui 241000 China
2. Rural Energy & Environment Agency, Ministry of Agriculture, Beijing 100125, China
3. Shenzhen GenProMetab Biotechnology Co., Ltd, Shenzhen, Guangdong 518101, China
4. Collaborative Innovation Center of Recovery and Reconstruction of Degraded Ecosystem in Wanjiang City Belt, Anhui Province, Anhui Normal University, Wuhu, Anhui 241000, China

#These authors contributed equally to this work.

* Corresponding author. E-mail address: ylxi1965@126.com (Y.-L. Xi)

Supplementary Table 1. Physical and chemical indices of the three sampling lakes.

Parameters	Lake Hui	Lake Tingtang	Lake Fengming
T (℃)	22	25	22.3
DO (mg/L)	10.24	7.5	9.26
pH	9.82	7.82	8.50
DTN (mg/L)	0.31	1.05	0.80
DTP (mg/L)	0.078	0.060	0.015
Chl–a (μg/L)	8.19	32.76	36.58
Al (mg/L)	0.375	BDL	0.033
As (mg/L)	0.212	BDL	BDL
Cd (mg/L)	0.002	BDL	BDL
Co (mg/L)	BDL	BDL	BDL
Cr (mg/L)	0.055	BDL	BDL
Cu (mg/L)	0.001	0.002	0.002
Fe (mg/L)	BDL	BDL	BDL
Mn (mg/L)	BDL	0.001	0.005
Mo (mg/L)	0.346	0.007	0.013
Pb (mg/L)	BDL	BDL	0.034
Se (mg/L)	0.164	BDL	BDL
Si (mg/L)	2.293	1.419	0.985
Zn (mg/L)	0.005	0.003	0.005
Hg (μg/L)	0.121	0.144	0.113

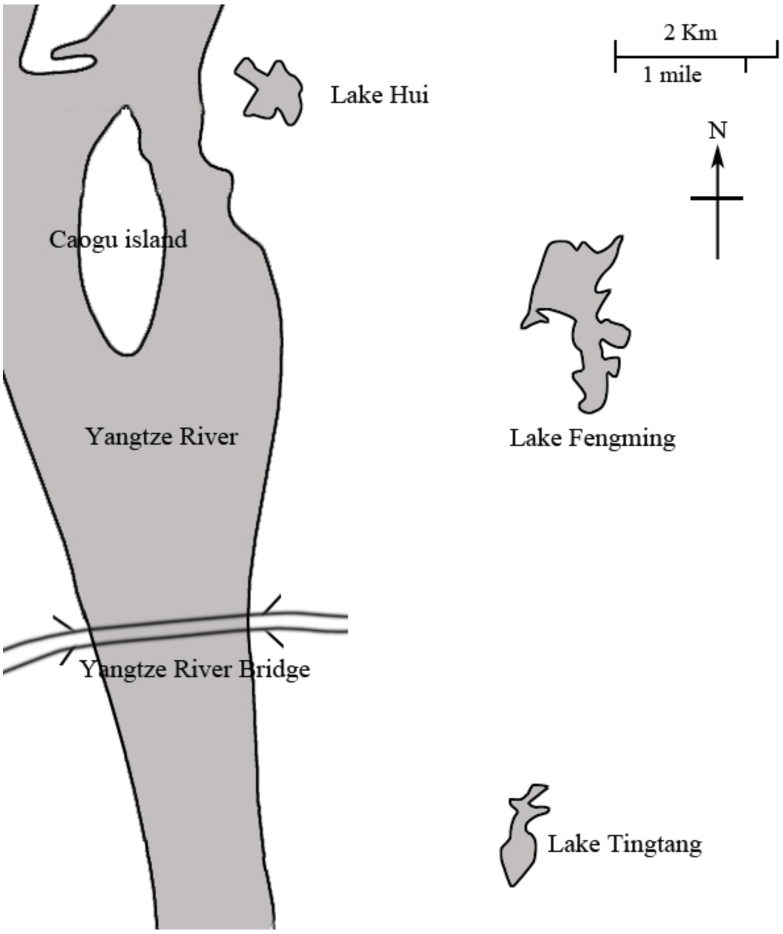

Supplementary Figure 1. Geographical locations of three sampling lakes. This map was hand-drawn based on the information from Google Earth.
